# Barriers and facilitators and the need for a clinical guideline for microbiological diagnostic testing in the hospital: a qualitative and quantitative study

**DOI:** 10.1007/s10096-019-03516-z

**Published:** 2019-03-07

**Authors:** Saskia J. Bogers, Frederike V. van Daalen, Sacha D. Kuil, Menno D. de Jong, Suzanne E. Geerlings

**Affiliations:** 1 Department of Internal Medicine, Division of Infectious Diseases, Amsterdam UMC, University of Amsterdam, Amsterdam, The Netherlands; 2 Department of Medical Microbiology, Amsterdam UMC, University of Amsterdam, Amsterdam, The Netherlands

**Keywords:** Antibiotic stewardship programmes, Clinical guidelines, Antimicrobial resistance, Microbiological diagnostic testing, Infectious diseases, Barriers and facilitators

## Abstract

**Abstract:**

The appropriate use of microbiological investigations is an important cornerstone of antibiotic stewardship programmes, but receives relatively limited attention. This study aimed to identify influencing factors in performing microbiological diagnostic tests and to assess the need for a clinical guideline. We performed a qualitative (focus group) and quantitative (online questionnaire survey) study among medical specialists and residents to identify physicians’ considerations in performing microbiological diagnostic tests and to assess the need for a diagnostic guideline. The questionnaire consisted of 14 statements, divided into three categories: knowledge, influencing factors and presence of guidelines. The questionnaire was sent to physicians of the departments of internal medicine, intensive care, paediatrics and pulmonology in five hospitals in the Netherlands. Sub-analyses for medical specialists versus residents and for paediatric versus non-paediatric departments were performed. We included 187 completed questionnaires in our analyses. The physicians reported having adequate knowledge on methods, time-to-result and accuracy, but inadequate knowledge on costs of the tests. Patients’ clinical condition, comorbidity, local guidelines and accuracy of tests were appraised as the four most important influencing factors to perform tests. Over 70% (132/187) of physicians reported being interested in a guideline for microbiological diagnostic testing. Fifteen physicians (8.0%) provided additional comments. This study identifies the influencing factors to microbiological testing and shows the demand for a clinical guideline among physicians.

**Importance:**

Microbiological diagnostic tests are an important cornerstone within antibiotic stewardship programmes [1–5]. These programmes aim to ameliorate the appropriate use of antibiotics and thus improve clinical outcomes of infectious diseases, whilst reducing the emergence of antimicrobial resistance [6]. However, inappropriate microbiological testing is a widely recognised problem [7–12], and influencing factors to testing have not been studied in the past. Our research shows the demand for a clinical guideline among physicians, and it identifies their influencing factors to testing. These results can be used to create a clinical guideline for microbiological diagnostic testing, thus supporting antibiotic stewardship programmes and reducing antimicrobial resistance.

## Introduction

Antibiotic stewardship programmes (ASPs) have been introduced in hospitals worldwide to guide the appropriate use of antibiotics with the aim of improving clinical outcomes of infectious diseases whilst reducing the emergence of antimicrobial resistance (AMR) and the risks of adverse reactions by limiting unnecessary exposure to antibiotics [6]. Microbiology laboratory diagnostics are an important cornerstone within these ASPs and crucial in guiding a clinically safe and cost-effective antimicrobial treatment [1–5]. However, the appropriate use of microbiological investigations as an important cornerstone of ASPs receives relatively limited attention.

Our group recently showed that the use of an antibiotic checklist for patients in need of antibiotic therapy significantly improved appropriateness of antibiotic use. However, it did not result in an increase in appropriate microbiological diagnostic testing from suspected sites of infection when indicated [13]. This might result from a lack of clear clinical guidelines for microbiological diagnostic testing in case of common infectious diseases such as community-acquired pneumonia (CAP) and urinary tract infections (UTI) [14, 15].

We hypothesise that a guideline for microbiological diagnostic testing will improve the appropriate use of such diagnostics to guide the appropriate use of antibiotics. The development of a useful and effective guideline requires understanding of the considerations, facilitators and barriers among physicians for the use of microbiological diagnostics in clinical management. Therefore, the aim of the current study was to identify the influencing factors in performing microbiological diagnostic tests and to assess the need for a clinical guideline for microbiological diagnostic testing for common infectious diseases.

## Materials and methods

We performed a qualitative (focus group) and quantitative study (online questionnaire survey) among medical specialists and residents to identify physicians’ considerations in performing microbiological diagnostic tests and to assess the need for a diagnostic guideline.

### Development of the questionnaire

Firstly, two researchers in the field of infectious diseases (F.D., S.K.) compiled a focus group guide including a comprehensive list of determinants that could influence the performance of microbiological diagnostic tests, based on previous research [16–18]. This list included guideline, professional and patient factors. Secondly, a multidisciplinary focus group was formed consisting of six medical specialists (one paediatrician, one pulmonologist, one intensive care specialist, one microbiologist, one infectious disease specialist and one acute internal medicine specialist) from a university hospital. This focus group discussed the list of determinants in a meeting of 90 min. For the design and conduction of this focus group meeting, internationally established guidelines for focus groups were used [19, 20]. The participants were selected purposively by the researchers and approached by email. The participants were informed of the goals of the focus group. Written informed consent was obtained from all participants and audio recording of the session was made. The focus group took place at the university hospital.

After the focus group meeting, we categorised the determinants that were identified as the most important influencers of the performance of microbiological diagnostic tests. Based on these categories, we developed a questionnaire.

The final questionnaire consisted of 14 statements. These statements could be divided into three categories: ‘knowledge on microbiological diagnostic tests’ (4 statements), ‘influencing factors’ (8 statements) and ‘diagnostic guidelines’ (2 statements). An overview of these statements is shown in Table 1. The level of agreement or disagreement with the statements in the questionnaire was measured by a 5-point Likert scale (1, ‘completely disagree’, and 5, ‘completely agree’). Additionally, physicians were invited to add comments in an open question at the end of the questionnaire.

**Table 1:** Content of questionnaire

Knowledge on microbiological diagnostic tests	
I have knowledge of accuracy (sensitivity/specificity/PPV/NPV) of various microbiological tests I order	
I have knowledge of methods (culture, PCR, serology, microscopy) of various microbiological tests I order	
I have knowledge of expected time to result of various microbiological tests I order	
I have knowledge of costs of various microbiological tests I order	
Influencing factors	
The clinical condition of the patient is an influencing factor on microbiological testing for me	
Comorbidity in a patient is an influencing factor on microbiological testing for me	
Accuracy of various microbiological tests is an influencing factor on microbiological testing for me	
Costs of various microbiological tests is an influencing factor on microbiological testing for me	
Workload is an influencing factor on ordering more microbiological tests for me	
Workload is an influencing factor on ordering less microbiological tests for me	
Faster time to result of various microbiological tests is an influencing factor on ordering tests for me	
Local guidelines influence my decision on microbiological testing	
Diagnostic guidelines	
There is a local guideline for microbiological diagnostics in my hospital (yes/no/do not know)	
I have interest in a clinical guideline for microbiological diagnostics for common infectious diseases	

Questions set out on a 5-point Likert scale (1, ‘totally disagree’, and 5, ‘totally agree’) unless otherwise indicated

*PPV* positive predictive value, *NPV* negative predictive value

The questionnaire was completed anonymously, but we asked for the participant’s function, department, hospital, sex and age. No characteristics were reported about the researchers. Questionnaire distribution and data collection were done using LimeSurvey version 2.6.7.

### Setting and participants

Five Dutch hospitals, including one university hospital and four non-university teaching hospitals, were included. Physicians from the departments of internal medicine, intensive care, paediatrics and pulmonology were invited to participate in the questionnaire. For each hospital, we had one contacting physician for the department of paediatrics, and one contacting physician for the departments of internal medicine, pulmonology and intensive care together. For the university hospital, we had a separate contacting physician for the intensive care department. These contacting physicians sent out the link to our questionnaire to all physicians from that department via email and informed us about the number of recipients in order to calculate response rates. The participating physicians included medical specialists and residents with various levels of professional experience, all of whom were involved in direct patient care.

### Analysis

We included questionnaires in the analyses if all statements were appraised. We calculated response rates by dividing the number of included questionnaires by the number of recipients of the questionnaire as reported by the contacting physicians.

For each of the four statements on knowledge on microbiological testing, the answers on the 5-point Likert scale were set out in a bar chart divided by group (medical specialists, residents, paediatrics department and non-paediatrics departments). The division between medical specialists versus residents was made to depict differences in professional experience. The division between the paediatrics department versus non-paediatrics departments was made to depict epidemiological, clinical and microbiological differences in infectious diseases and because separate diagnostic and treatment guidelines exist for the two [21–25].

For the eight statements on influencing factors, we used a different approach to analyses because we were most interested in which factors were more and which were less of an influence on microbiological testing. We calculated the means of the 5-point Likert scale (1, ‘completely disagree’, and 5, ‘completely agree’) and ranked them according to the level of agreement. A mean outcome to a statement over 3 (neutral) was considered to be agreed upon.

The answers to the final statement (‘I have interest in a clinical guideline for microbiological diagnostics for common infectious diseases’) are depicted on a 5-point scale histogram.

We categorised the comments added by the participants. If comparable comments were mentioned three times or more, the comment was considered to be relevant.

#### Datas availability

The datasets used and/or analysed during the current study are available from the corresponding author on reasonable request.

## Results

### Participants and response rates

In total, 640 physicians received our questionnaire, ranging between 17 and 185 per hospital. We received 201 responses, of which 187 were fully completed. These 187 responses were included in the analyses. The overall response rate was 29.2% (187/640). The number of completed questionnaires per hospital ranged from 9 to 111, with a response rate ranging between 24.4 and 52.9% per hospital. The response rate was higher for non-paediatric departments (133/420; 31.7%) than for paediatric departments (54/220; 24.6%). Most participants were medical specialists (106/187; 56.7%), and most participants were working in the department of internal medicine (103/187; 55.1%). The participant characteristics are summarised in Table 2.

**Table 2:** Participant characteristics

Participant characteristics (*N* = 187)	Number (percentage) or mean (± SD)
Physician type	
Resident	81 (43.3)
Medical specialist	106 (56.7)
Medical department	
Intensive care	24 (12.8)
Internal medicine—infectious disease specialist	12 (6.4)
Internal medicine—non-infectious disease specialist	91 (48.7)
Pulmonary medicine	6 (3.2)
Paediatrics—infectious disease specialist	2 (1.1)
Paediatrics—non-infectious disease specialist	52 (27.8)
Hospital	
Hospital 1 (university hospital)	111 (59.4)
Hospital 2 (teaching hospital)	30 (16.0)
Hospital 3 (teaching hospital)	9 (4.8)
Hospital 4 (teaching hospital)	24 (12.8)
Hospital 5 (teaching hospital)	13 (7.0)
Age (years)	40.6 (10.6)
Sex	
Male	73 (39.0)
Female	111 (59.4)

*SD* standard deviation

### Knowledge on microbiological diagnostic tests

**Fig. 1:**
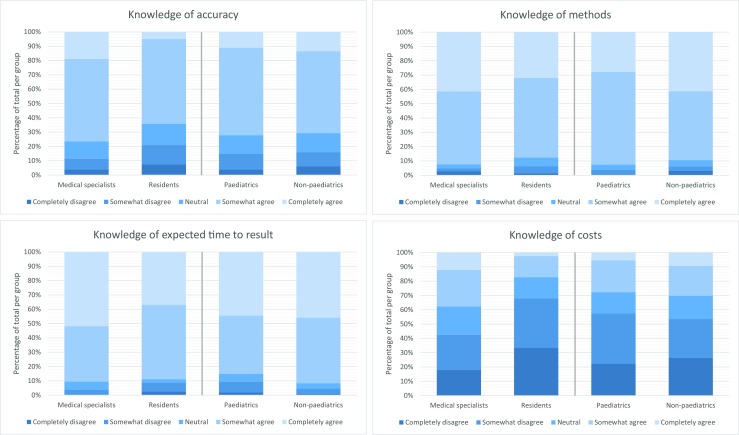
Knowledge on microbiological diagnostic tests per group based on four statements: (a) I have knowledge of accuracy (sensitivity/specificity/PPV/NPV) of various microbiological tests I order. (b) I have knowledge of methods (culture, PCR, serology, microscopy) of various microbiological tests I order. (c) I have knowledge of expected time-to-result of various microbiological tests I order. (d) I have knowledge of costs of various microbiological tests I order

Four statements about the knowledge on microbiological diagnostic tests were tested. Most physicians reported having adequate knowledge on methods (culture, PCR, serology, microscopy) and expected time-to-result of various microbiological tests. The vast majority of physicians also reported adequate knowledge on accuracy (sensitivity/specificity/positive predictive value/negative predictive value) of various microbiological tests. However, only a minority of physicians reported having adequate knowledge of the costs of various microbiological tests. For all statements, reported knowledge was higher in medical specialists and those working in non-paediatric departments compared with residents and those working in paediatric departments (Fig. 1).

### Influencing factors

**Fig. 2:**
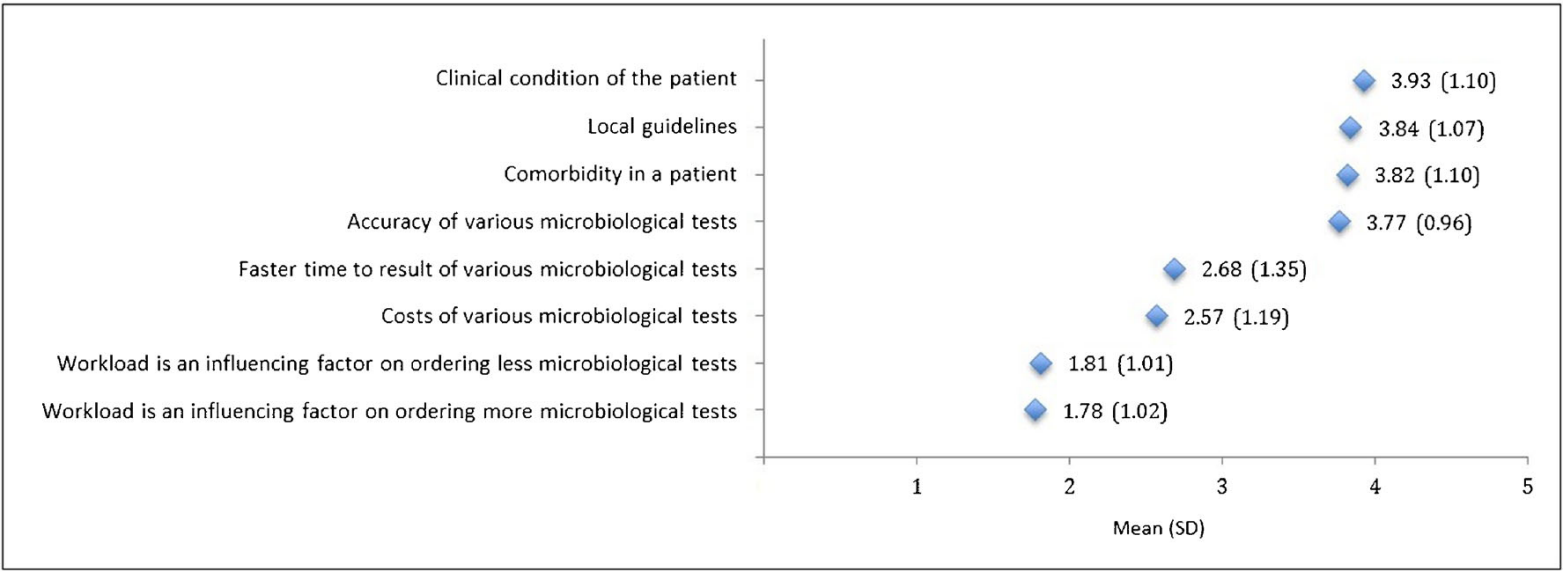
Rank order of importance for the eight statements on influencing factors in performing microbiological diagnostic tests. 1, ‘totally disagree’, and 5, ‘totally agree’, on the 5-point Likert scale. SD, standard deviation

Eight statements on influencing factors in performing microbiological diagnostic tests were tested. Four were agreed upon to be important factors (i.e. had a mean over 3 (neutral) on the Likert scale), namely the clinical condition of the patient, the availability of a local guideline, the comorbidity of the patient and the accuracy of various microbiological tests. The other four statements were disagreed upon, namely workload as a factor to order more diagnostic tests, workload as a factor to order less diagnostic tests, costs of various tests and faster time-to-result of tests (Fig. 2).

### Diagnostic guidelines

In total, 122/187 (65.2%) physicians reported that local guidelines in performing microbiological diagnostic tests were available in their hospital. Fifty-one physicians (27.3%), of whom 18 (35.3%) were medical specialists and 33 (64.7%) were residents, stated they did not know if such guidelines exist in their hospitals. A total of 12 (23.5%) were working in the paediatrics versus 39 (76.5%) in the non-paediatric departments.

**Fig. 3:**
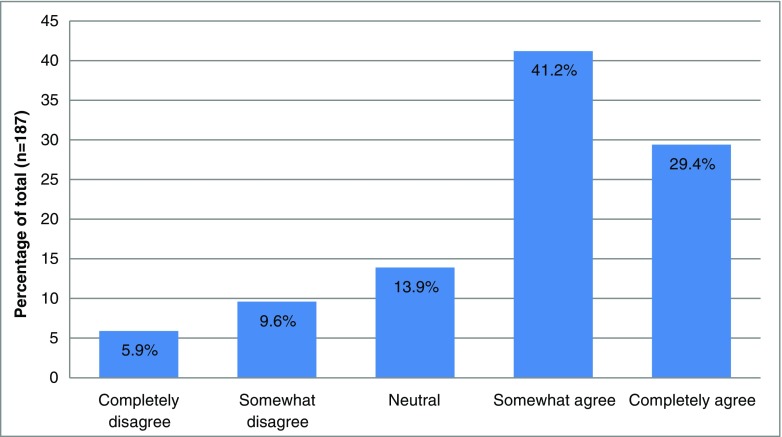
Total reported interest in a clinical guideline for microbiological diagnostic tests for common infectious diseases

Most physicians (132/187; 70.6%) reported being interested in the availability of a clinical guideline for microbiological diagnostic tests for common infectious diseases (Fig. 3). Of all participating residents, 71.8% (59/81) were interested in the clinical guideline. Of all participating medical specialists, 68.9% (73/106) were interested. Of all participants from paediatrics departments, 74.1% (40/54) were interested in the clinical guideline. Of all participants from non-paediatric departments, 69.2% (92/133) were interested.

### Participants’ comments

Fifteen physicians (8.0%), out of whom 67% (10/15) were from non-paediatric departments and 60% (9/15) were medical specialists, provided additional comments. Five physicians stressed logistical barriers as an important influencing factor for microbiological testing. They specifically mentioned time-pressure, insufficient stock of appropriate tests in the emergency department (ER) or ward and slow time-to-result of microbiologic tests. Four physicians stressed the need for better understanding of microbiologic testing, either for themselves or for (young) colleagues and (ER) nurses, and stated that this lack of knowledge was the biggest barrier for correct use of testing. Three of the participating physicians stated that they would find a checklist or guideline that also mentions costs, time-to-result, diagnostic value and accuracy of various tests essential to make well-informed decisions in performing these microbiological diagnostic tests.

## Discussion

In this study, we aimed to create an overview of barriers and facilitators in performing microbiological diagnostic tests for infectious diseases in general and to determine the need for a diagnostic guideline for infectious diseases. We identified patient characteristics and the existence of local guidelines as the most important influencing factors in performing microbiological diagnostic tests for physicians. Workload was the least important influencing factor. Most physicians reported to have adequate knowledge on technical details about various microbiological tests, but reported to have inadequate knowledge of the costs of these tests. Over 70% (132/187) of the participating physicians expressed interest in the development of a clinical guideline for microbiological testing.

Physicians reporting to have adequate knowledge on microbiological diagnostic tests were mostly medical specialists (versus residents) and working in non-paediatric departments (versus paediatric departments). However, the same group also reported being interested in the availability of a clinical guideline for microbiological diagnostic tests the most.

One previous study also reported that diagnostic testing is influenced by patient characteristics [26]. To our knowledge, no other studies describe influencing factors to physicians in performing microbiological diagnostic tests. On the other hand, numerous studies report both over- and underuse of diagnostics in various clinical settings [7–12], supporting our notion that an evidence-based guideline is needed. Our finding that most physicians reported to have adequate knowledge on technical details of tests is notable; one of the outcomes of the focus group was that there is a lack of knowledge on the various techniques, timing and interpretation of microbiological diagnostic tests. This might be due to participants giving socially desirable answers, although participants did report a poor knowledge of the costs of microbiological tests. Concerning knowledge, previous studies suggest that education on antimicrobial stewardship needs to be expanded and started at the beginning of the education, which is the time when education and other influences determine future behaviour the most [27, 28]. Based on our results, we recommend that education on the appropriate use and costs versus benefits of microbiological testing as a diagnostic part of the antibiotic stewardship programme be added to the education of all medical students.

Our study has several strengths. To our knowledge, this is the first study to identify factors that influence physicians’ considerations to perform microbiological diagnostic tests. It uses both qualitative (focus group) and quantitative research (online questionnaire survey) approaches. We used a focus group, an effective tool for qualitative research [29–31], consisting of representatives from different medical disciplines to develop the questionnaire. The questionnaire was developed based on validated measurement instruments for identifying determinants of innovations [16, 17]. Furthermore, we invited physicians with various levels of professional experience and from various departments to participate ensuring a wide scope of perspectives.

The most important limitation of our study is the wide range in response rates in the different hospitals. This is possibly due to the fact that in some hospitals, the contacting physicians were more motivated to have their departments participate in the study and put out more reminders to complete the questionnaire compared with others, or the presence of many other questionnaires for the same target group at the same time. A lack of motivation to complete our questionnaire might be explained by the fact that evidence in performing microbiological diagnostic tests is not a key focus point for physicians, since more research has been focussed on antibiotic treatment compared to diagnostic methods. Second, our chosen method of recruitment poses some limitations; we emailed the contacting physicians a link to the questionnaire with the request to forward it to the target groups. We aimed to invite as many physicians as possible to participate with this approach, but the downside is that the exact number of physicians per subgroup that received the link to the questionnaire was unclear and, consequently, it was impossible to determine the response rates per subgroup. Based on an analysis in one hospital where we could retrieve exact information on the number of invitations sent to the subgroups, we determined the response rate to be 20.7% (6/29) among medical specialists and 29.2% (7/24) among residents. As these response rates are also found in other studies [32, 33], we assume the response rates per subgroup to be similar for the other hospitals. Furthermore, the generalisability of our study might be limited as we only invited non-surgical departments to participate. A future study could focus more on surgical specialties to test if the outcomes in terms of barriers and facilitators to microbiological testing, knowledge of tests and need for a diagnostic guideline would differ from the currently tested groups. In our study, we included infectious disease specialists; this might have led to bias in knowledge on various microbiological tests as we hypothesise that this knowledge is less in other specialties. However, our group still expresses a gap in knowledge and a desire for a guideline, and we consequently expect this effect to be even bigger in departments that are less trained in infectious diseases. Further follow-up research might also differentiate between various infectious diseases to determine if the results found in this study differ between the most common infectious diseases to gain more specific insights to the current barriers and facilitators, knowledge and need for a diagnostic guideline.

As previously stated, the inappropriate use of microbiological diagnostic tests is widely recognised as a problem, but influencing factors in performing these tests remained largely unclear. The influencing factors identified in this study are a good starting point for improvement and can be used in the development of clinical guidelines. Most participants stated that local guidelines in performing microbiological diagnostic tests are available in their hospitals and that this availability is one of the most important influencing factors in performing microbiological diagnostic tests. However, this study also shows that there is an interest in clinical guidelines for microbiological diagnostic testing for common infectious diseases. This discrepancy is likely due to the fact that only local guidelines concerning empirical antimicrobial treatment, but not about microbiological testing, were available in the participating hospitals. An additional factor could be that the current local guidelines lack consideration of the influencing factors identified in this study. To our knowledge, no local or national diagnostic guidelines exist for infectious diseases. Moreover, the guidelines for specific infectious diseases that do exist state to perform microbiological diagnostic tests but they do not list the factors that should be taken into account when deciding to test and they do not take physicians’ barriers and facilitators to testing into account. The discrepancy that the subgroups who reported to have the most adequate knowledge on microbiological diagnostic tests are the same subgroups that report being interested in the availability of a clinical guideline could indicate that there is a knowledge gap in the importance of the appropriate use of microbiological investigations and antibiotic stewardship programmes.

In conclusion, our study indicates that there is a need among clinicians for clinical guidelines for microbiological diagnostic testing for common infectious diseases. The most important influencing factors in performing microbiological diagnostic tests identified in this study, namely the clinical conditions and comorbidities in a patient, should be included in these guidelines. Finally, education on microbiological diagnostic testing should be included in the early stages of medical education and training. Together, these interventions will promote the appropriate use of microbiological investigations and thus should be the next step in our efforts to support antibiotic stewardship programmes and to reduce the emergence of antimicrobial resistance.
